# Antimicrobials Affect the Fat Body Microbiome and Increase the Brown Planthopper Mortality

**DOI:** 10.3389/fphys.2021.644897

**Published:** 2021-03-15

**Authors:** Jiateng Shi, Yang Song, Xuping Shentu, Xiaoping Yu

**Affiliations:** Zhejiang Provincial Key Laboratory of Biometrology and Inspection & Quarantine, College of Life Science, China Jiliang University, Hangzhou, China

**Keywords:** *Nilaparvata lugens*, antimicrobials, fat body, microbiome, mortality

## Abstract

Symbionts in the abdomen fat body of brown planthopper (BPH) play an important role in the growth and reproduction of their host, *Nilaparvata lugens* Stål (Hemiptera: Delphacidae). Thus, controlling BPH infection on rice by inhibiting symbionts with antimicrobials is feasible. However, the effect of antimicrobials on the microbiome in the fat body and the relationship between microbial community and mortality have not been fully elucidated. A decrease in the total number of yeast-like symbiotes in the fat body and elevated mortality were observed after exposure to toyocamycin, tebuconazole, and zhongshengmycin. Additionally, we found that the antimicrobials reduced bacterial diversity and increased fungal diversity in the fat body and altered the bacterial and fungal community structure. Although the total absolute abundance of bacteria and fungi decreased after antimicrobial exposure, the absolute abundance of *Serratia* increased, indicating that *Serratia*, which was the most dominant in the fat body, is an important symbiont involved in resistance to antimicrobials. After antimicrobial exposure, seven genera, which probably participated in the nutrition and development function of the host, were totally eliminated from the fat body. Overall, our study enriches the knowledge of microbiomes in the fat body of BPH under antimicrobial treatment and the disturbance of symbionts would be further used to help other pesticides to control pests.

## Introduction

The brown planthopper (BPH), *Nilaparvata lugens* Stål, is one of the typical monophagous pest herbivores of rice in Asia, which feeds on sap from rice plant phloem through its mouthpart and causes hopper burn when BPH population is high (Cheng et al., 2013). The BPH harms rice plants by transmitting viruses, such as the rice ragged stunt virus and rice grassy stunt virus (Bao and Zhang, 2019). Therefore, the BPH is one of the significant pests affecting rice production, and *N. lugens* outbreaks have led to huge rice yield losses since 1970s in many East and Southeast Asian countries (Wu et al., 2018). However, the BPH is hard to control due to its long-distance migration characteristic and resistance to rice varieties and pesticides. Recent studies showed that the ability to migrate and resistance to external environmental conditions is related to the abundance and variability of endosymbionts (Tang et al., 2010; Malathi et al., 2018).

The BPH harbors various endosymbionts, which are dispersed in its gut and hemolymph or within its fat body cells as mostly hemipteran insects (Zhang et al., 2019). The BPH has been shown to establish a close symbiont relationship with microbes; the BPH provides habitat for endosymbionts (Cheng and Hou, 2001), and the endosymbionts in turn contribute to the nutrition, growth, development, and acclimatization evolution of the BPH and especially, to BPH reproduction (Xue et al., 2014). Among all the microorganisms BPH harbors, yeast-like symbiotes (YLS) and symbiote bacteria in the fat body can synthesize essential amino acids, steroids, and vitamins, maintaining host nutrition supply and provide vital physiological and trophic functions for the growth and reproduction of BPH (Xue et al., 2014; Bao and Zhang, 2019). Therefore, in the control of the spread and outbreak of BPH for the rice planting industry, microbes in the fat body of the BPH have been considered a crucial target in biological pest management.

Regarding the fungal community, several strains have been identified through different analysis methods. For example, *Hypomyces chrysospermus* was identified from the fat body by centrifugation and 18S rRNA sequences (Noda et al., 1995), *Candida lipolytica* and *Sterigmatomyces halophilus* were identified from the eggs using 26S rDNA sequences (Zhang and Fang, 2001; Zhang et al., 2007), *Saccharomycetales* sp., *Debaryomyces hansenii*, and several uncultured fungi were detected from the fat body in the abdomen using novel methods, such as nested PCR-denaturing gradient gel electrophoresis (Hou et al., 2013). Meanwhile, the bacterial community was identified from female adult through 16S rRNA sequencing, and 18 bacterial symbionts in four phyla were identified in *N. lugens* (Tang et al., 2010). Although many strains in the fat body have been identified, the high-throughput sequencing based on internal transcribed spacer (ITS) and 16S rRNA sequences can provide in-depth microbiome information and some microbes with low abundance could also be detected (Shi et al., 2010). Moreover, the function of endosymbionts in the fat body has been studied by metagenome sequencing and pathway analysis (Fan et al., 2015, 2016), but these studies regard the endosymbionts as a whole and focus on the function of total endosymbionts. So it is meaningful to alter the endosymbiont community though the treatment of antimicrobials, and reveal the relationship between the genera of microbiome and the phenotypic changes (such as the mortality).

In this study, we preliminarily investigated mortality under different types of antimicrobials, including the toyocamycin, tebuconazole, and zhongshengmycin. These three antimicrobials were selected from a pre-test of seven kinds of antimicrobials, which has the top three mortality of BPH after treatment of 4 days (data not shown). These three kinds of antimicrobials have different mechanism to the microbes. Toyocamycin is a nucleoside antibiotic and generated by the fermentation of *Streptomyces diastatochromogenes* 1,628. It exhibits both antibacterial and antifungal activities and effective against pathogenic fungi, including *Rhizoctonia solani* and *Fusarium oxysporum* (Fan et al., 2020). Tebuconazole is a broad-spectrum triazole fungicide and causes DNA damage and induces the mitochondrial apoptotic pathway in the fungal pathogens of plants (Muñoz-Leoz et al., 2011), which could affect the fungus in the fat body. Zhongshengmycin is a new kind of antimicrobial and is used in 10 provinces in China and some other Southeast Asian regions for the control of plant diseases. Zhongshengmycin can inhibit *Xanthomonas oryzae* pv. oryzae (Jiang et al., 2003), so the zhongshengmycin could lead to the change of bacterial community. Although all three kinds of antimicrobials can be used in controlling pathogens in plants, antimicrobials can affect the microbiome in the fat body by feeding behavior and influence the mortality of BPH.

The microbe community of bacteria and fungi in the fat body after exposure to different antimicrobials was determined by sequencing the 16s rRNA and through ITS. We evaluated the relationship between microbe community composition and antimicrobial types. Finally, the absolute change in microbes was monitored using quantitative PCR (qPCR) for the characterization of the microbial community. This study represents the first experimental research on different microbial structures of the fat body under exposure to different antimicrobials, which could be used to assist pesticides to control pests.

## Materials and Methods

### Insect Rearing and Mortality

The BPH population used in this study was originally collected from rice fields in Hangzhou (E120°12, N30°16) and maintained on the susceptible rice variety TN1 at 26 ± 1°C, 70–80% relative humidity, and 16 h light:8 h dark photoperiod in a climate chamber. The rice TN1 was grown on soil from a local farm in the same conditions as those used in BPH rearing.

Antimicrobials were sprayed during rice tillering with a mini-sprayer at recommended concentrations on the China Pesticide Information network and preliminary experiment results [Toyocamycin (100 mg/L), tebuconazole (125 mg/L), and zhongshengmycin (60 mg/L)]. The three stems of sprayed rice plants were placed in a test tube (2.5 cm in diameter and 30 cm in height) filled with 20 ml rice nutrient solution, and then 30 emergence 1-day old adults were placed in the test tubes, which were covered with two layers of gauze. The tubes were placed in a greenhouse at 26°C with 16 h light:8 h dark photoperiod under an artificial light. Ten replications were conducted in the whole experiment, and water treatment was used as the control. Five replications were used to calculate the mortality and another five replications were used to account the symbionts in the fat body. The dead number of BPH was counted at day 1, 2, and 4. The accumulated mortalities were calculated according to the following Eq. (1)

$$A=\frac{\sum_{1}^{n}\;X}{30}$$ (1)

Where *A* is the accumulated mortalities, *n* is the day, *x* is the death number of BPH, 30 is the total number of emergence 1-day old adult BPH. The value is the mean ± SE, the different letters on the top of each bar indicate that the data are significantly different by Tukey test for multiple comparisons which is analyzed with SPSS, version 25.0 (Chicago, IL, United States).

### Symbiont Accounting

The number of YLSs was counted with a hemocytometer by the morphological characterization. The bacteria are excluded because the bacteria are too small in the field of microscope to identify or count. In addition, the number just showed the total number of YLSs without distinguishment of genera or species. Three of the adult BPH samples at the day 4 after different treatment conditions were collected from each test tube and sterilized using 75% ethanol and washed twice with sterilized water. Then, the wings of the BPH were removed by sterilized tweezers, and the head, thorax, and legs were placed in 50 μl of sterilized PBS buffer in 1.5 ml Eppendorf tube, and the BPH was blended with the sterilized plastic grinding rod until no obvious tissue could be seen. The PBS were sterilized with the membrane filtration to avoid the contamination of microbes, and the PBS could dilute the body fluid of BPH, which could much easier to extract and fulfill the hemocytometer. The solution was added to the hemocytometer, and strain number was determined under a binocular optical microscope (400×). Each sample was accounted three times, and five replications were carried out. The value was the mean ± SE, the different letters on the top of each bar indicated that the data were significantly different by *t*-test after ANOVA which was analyzed with SPSS, version 25.0 (Chicago, IL, United States).

### Fat Body Dissection Collection and DNA Preparation

After treatment with antimicrobials for 4 days, the BPH was collected, surface-sterilized in 75% ethanol for 60 s, and rinsed with sterile water three times. Under a stereomicroscope, the BPHs were put on a clean Petri dish, and the head, wing, legs, and thorax was removed together by separating the thorax and abdomen. After the abdomen was dissected with forceps, the organ, ovary, and cuticle were removed, and then the left oil-like part is the abdominal fat body. The fat body from 100 BPH was pooled in a centrifuge tube as a replication, and each antimicrobial treatment was performed in three replicates. Microbial genomic DNA was extracted using a FastDNA SPIN kit for soil (OMEGA, Bio-Tek, Georgia, United States) according to the manufacturer’s protocol. DNA quantity and quality were measured on a NanoDrop 2000 spectrophotometer (Thermo Fisher Scientific, United States) and 1.0% agarose gels. All DNA samples were preserved at −20°C before PCR analysis.

### PCR Amplification and High-Throughput Sequencing

The V3-V4 variable region of the bacterial 16S rRNA gene was amplified with primers 338F (5'-ACTCCTACGGGAGGCAGCAG-3') and 806R (5'-GGACTACHVGGGTWTCTAAT-3'). The ITS of the fungal ribosomal operon was amplified with universal primers ITS3F (5'-GCATCGATGAAGAACGCAGC-3') and ITS4R (5'-TCCTCCGCTTATTGATATGC-3') for fungal diversity analysis (Takahashi et al., 2014; Waud et al., 2014). A 20 μl PCR reaction mixture containing 4 μl of 5 × FastPfu Buffer, 2 μl of 2.5 mM dNTPs, 0.4 μl of FastPfu polymerase, 0.2 μl of BSA, 10 ng of template DNA, and 0.8 μl of each primer, was added to ddH_2_O to a final volume of 20 μl. The following PCR cycle conditions were used: initial denaturation at 95°C for 3 min; followed by 27 cycles of 30 s at 95°C, 30 s at 55°C, and 45 s at 72°C; and final extension for 10 min at 72°C. Before high-throughput sequencing, the concentrations of all purified PCR amplicons were examined through 2% agarose gel electrophoresis and quantified with NanoDrop 2000. Then, 10 ng of the 16s rRNA gene amplicons and 10 ng of ITS gene amplicons were pooled and subjected to MiSeq sequencing at MajorBio Techonology Co., Ltd., Shanghai, China.

### Bioinformatic and Statistical Analysis

Paired-end reads were merged using FLASH set to a minimum overlap of 10 bp and a mismatch rate lower than 0.02.^1^ The initial 16S rRNA and ITS gene sequences were processed, and demultiplexing was performed using the open-source Quantitative Insights into Microbial Ecology (QIIME) toolkit (version 1.8.0). Sequences that presented mismatches in the primer sequences; ambiguous bases; or chimeric sequences were discarded. Statistical analyses were performed using the UPARSE pipeline of USEARCH, and high-quality sequences were clustered into operational taxonomic units (OTU) with 97% similarity. The representative OTU identified based on the highest relative abundance in each OTU was selected for the construction of the overall OTU table. Then, taxonomic identification was performed using an RDP classifier and data sets from the SILVA database as 16S rRNA and fungal ITS reference under a confidence threshold of 70%. The raw data have been submitted to the National Center for Biotechnology Information Sequence database under the accession number SRR13356000-SRR13356047.

Alpha diversity was calculated using QIIME v1.8.0, which includes tools for creating a Venn diagram and determining Chao1 and Simpson indices, an abundance-based coverage estimator (ACE), and a Shannon estimator. Rarefaction curves were analyzed using the “alpha_rarefaction.py” script in QIIME. The ACE and a nonparametric richness estimator based on the distribution of singletons and doubletons (Chao1) were used in estimating species richness, and diversity indicators (Shannon and Simpson indices) were calculated and used in showing species diversity in all the samples. A large Shannon index means high species richness, whereas a high Simpson index indicated a large composition of a specific species.

Beta diversity was examined through principal coordinate analysis (PCoA) with the Bray-Curtis distance algorithm and used in showing difference in community structure among the samples. Compositional differences were examined using ANOSIM with 999 permutations and the Vegan Package. Significant differences among the groups were calculated using the nonparametric *t*-test, which was performed on Metastats. A value of *p* < 0.05 was considered significant.

### Absolute Microbial Abundance

The total copy number of bacterial and fungal community was calculated by the qPCR. The absolute abundance of each strain was estimated on the basis of relative abundance. The primers used in qPCR were the same as the sequencing primers. The reactions were carried out in three parallel samples, and TB Green Premix Ex Taq (Takara, Dalian, China), 0.4 μl of 10 μM primers, 0.4 μl of ROX reference dye, 6.8 μl of H_2_O, and 2 μl of templates were used. The PCR conditions were as follows: initial denaturation at 95°C for 30s; followed by one PCR 40 cycles at 95°C, 5 s, 60°C 30 s in the real-time PCR system. Absolute copy number was calculated from the standard curve (Supplementary Figure S1) generated by the amplification of target genes in a pGEM-T vector (Sangon Biotech, Shanghai, China).

## Results

### Mortality Under the Antimicrobial Exposure

**Figure 1:**
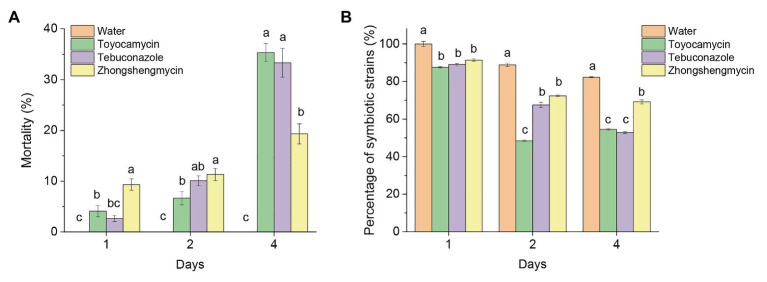
Effect of antibiotics on the mortality and number of strains in BPH. **(A)** Mean mortality (±SE) of adult BPH after the treatment of three antimicrobials on days 1, 2, and 4. **(B)** Effect of antimicrobials on the total number of yeast-like symbionts (YLSs) in the fat body, except the head, legs, and thorax, the number of YLSs in the control group at the day 1 was regarded as 100%. Values are mean ± SE, where *n* = 5. Different letters on the top of each bar indicate that the data are significantly different by Tukey test for multiple comparisons with a significance threshold of *p* < 0.05. The control group was treated with water.

The mortality of the BPH subjected to antimicrobial treatment increased constantly from 1 to 4 days after treatment (Figure 1A). In the first 2 days, the mortality of BPH treated with zhongshengmycin was higher than the BPH treated with toyocamycin and tebuconazole. The death rates of BPH treated with tebuconazole and toyocamycin exceeded that of BPH treated with zhongshengmycin at the 4th day. The highest mortality reached 35.67% after toyocamycin treatment, followed by 33.13%, which was obtained after tebuconazole treatment, at the 4th day.

Although the total number of YLSs in the control group decreased after emergence, symbionts subjected to antimicrobial treatment were reduced in number more quickly than those subjected to water treatment in the first 4 days (Figure 1B). At the 4th day, the number of symbionts in the control group was above 82%, but the microbes decreased to 54.49, 52.83, and 69.21% after toyocamycin, tebuconazole, and zhongshengmycin treatments, respectively. The results showed that the toyocamycin and tebuconazole groups had the lowest amounts of YLS and the highest mortality rates at the 4th day, indicating that microbial number in the host is related to insect mortality.

### Antimicrobials Affect Microbial Community Evenness

To investigate the effect of antimicrobials on the microbial community and its structure in the fat body of *N. lugens*, we sequenced the 16S rRNA gene V3-V4 variable regions and ITS variable regions with an Illumina MiSeq platform (Table 1). After initial quality control, an average of 35,029 high-quality sequences was obtained from 12 samples, and a total of 255 and 188 OTUs were obtained from the bacterial and fungal samples on the basis of 97% sequence similarity. The control group had the highest number of OTUs in the bacterial and fungal communities (195 OTUs for bacteria and 77 OTUs for fungi). The toyocamycin groups had the lowest OTUs in the bacterial (90 OTUs) and fungal communities (69 OTUs). Good’s coverage in all the 12 samples (three replicates for four treatments) were over 99%, and the rarefaction curves reached saturation plateau, suggesting that the current sequencing database facilitates the analysis of dominant microbial community information.

**Table 1:** Microbial community alpha-diversity characteristics in brown planthopper (BPH) under different antimicrobials.

Sample	OTUs	Phylum	Genus	Shannon	Simpson	Chao1	ACE
Bacterial community							
Water	195	12	127	2.14	0.20	138.65	157.97
Toyocamycin	90	6	58	1.29	0.44	90.77	104.93
Tebuconazole	90	9	60	1.54	0.36	86.21	106.48
Zhongshengmycin	114	11	83	1.53	0.34	109.79	153.44
Fungal community							
Water	77	6	33	0.52	0.79	35.23	35.51
Toyocamycin	69	6	19	0.70	0.74	53.43	54.26
Tebuconazole	72	5	16	0.69	0.72	43.03	44.60
Zhongshengmycin	76	5	11	0.71	0.72	46.33	42.32

In the bacterial community, 44 OTUs were shared by the four groups. Only 99, 10, 10, and 22 OTUs were found in the water, toyocamycin, tebuconazole, and zhongshengmycin groups, respectively (Supplementary Figure S2). The high Shannon index and low Simpson index in the control group suggested that antimicrobial treatment reduced bacterial diversity. An opposite effect was observed in the fungal community. The Chao1 index in the antimicrobial treatment groups were lower than that in the control group, showing that species richness decreased after antimicrobial exposure (Table 1).

In the fungal community, 21 OTUs were found in the four groups, and 28, 18, 27, and 31 OTUs were found the water, toyocamycin, tebuconazole, and zhongshengmycin groups, respectively. The Shannon, Simpson, and Chao1 indices were used in evaluating the diversity and richness of the microbial community. The Shannon index in the control group was lower than that in the antimicrobial treatment group, and the Simpson index in the control group was higher than that in the antimicrobial treatment group, indicating that the exposure of antimicrobials increased the complexity of the fungal community. The similar value of Chao1 index indicated that microbial richness of species was similar in all treatment (Table 1).

### Antimicrobial Treatment Alters Community Structure

**Figure 2:**
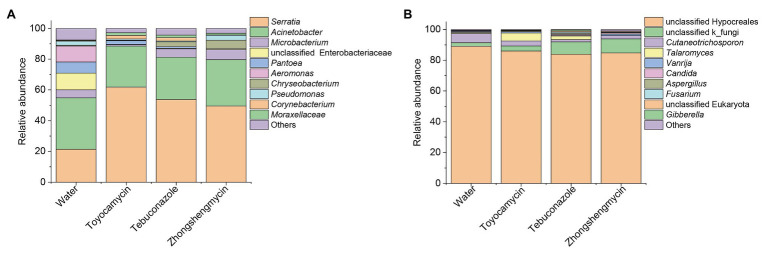
Relative abundances of top 10 bacteria **(A)** and fungi **(B)** with respect to relative abundance at the genus level in BPH. Each bar or column corresponds to a genus (three replications were high-throughput sequencing for each treatment). The value is the average of the relative abundance for the three replications calculated by SPSS 25.0 and the figures were drawn by Origin 8.5.

The bacterial and fungal genera between the control and antimicrobial treatment groups were analyzed from the taxonomic assignment performed at different levels of classification. At the genus level, 10 main genera were obtained in the four groups and had abundances above 1%. As for the bacterial community, 127, 58, 60, and 83 genera were obtained from the water, toyocamycin, tebuconazole, and zhongshengmycin groups, and 38 genera were shared by all the samples. *Serratia* and *Acinetobacter* were the two most predominant bacteria and composing 54.92, 88.48, 81.24, and 79.64% under the exposure of water, toyocamycin, tebuconazole, and zhongshengmycin, respectively. As for the fungal community, the genera obtained from the control, toyocamycin, tebuconazole, and zhongshengmycin groups were 33, 19, 16, and 11. Five main genera were shared by the four groups, namely, unclassified Hypocreales, unclassified k_fungi, *Cutaneotrichosporon*, *Talaromyces*, and *Vanrija*. Among all the fungal genera, Hypocreales genera were dominant in the four groups (Figure 2).

**Figure 3:**
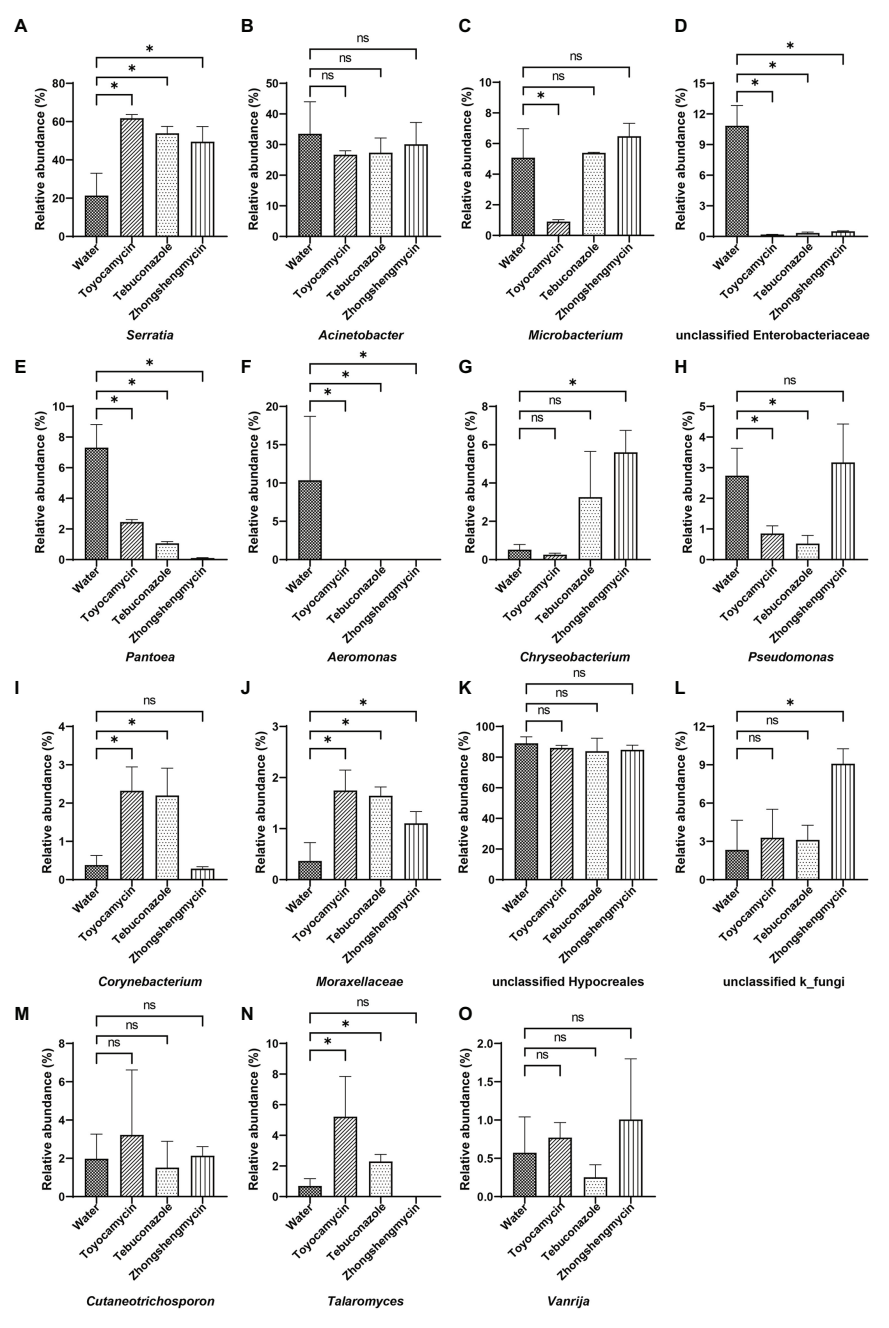
Comparative analysis of microbial abundance in dominant genera. **(A–J)** Comparative analysis of difference in bacterial abundance on day 4 in *Serratia*, *Acinetobacter*, *Microbacterium*, unclassified Enterobacteriaceae, *Pantoea*, *Aeromonas*, *Chryseobacterium*, *Pseudomonas*, *Corynebacterium*, and *Moraxellaceae*. **(K–O)** Comparative analysis of difference in fungal abundance on day 4 in the genera of unclassified Hypocreales, unclassified k_fungi, *Cutaneotrichosporon*, *Talaromyces*, and *Vanrija*. ns, no significant difference between the control group and the treatment groups at *p* < 0.05 levels. ^∗^Significant difference between the control group and the treatment groups at *p* < 0.05 levels.

The microbiome was analyzed under the four different treatments for the comparison of microbial compositions. Obvious difference in microbial richness was observed at a relative abundance of over 0.1% was selected and visualized using a bar chart (Figure 3). In the bacterial community, the top 10 bacterial genera (with relative abundances of >0.1%) had relative abundances of 92.5% in all the samples. The top 10 genera were as follows: *Serratia*, *Acinetobacter*, *Microbacterium*, unclassified *Enterobacteriaceae*, *Pantoea*, *Aeromonas*, *Chryseobacterium*, *Pseudomonas*, *Corynebacterium*, *Moraxellaceae*, and others. *Serratia* had the highest relative abundance, which was significantly affected by antimicrobial treatment. The relative abundance increased from 21.39 to 61.78, 53.87, and 49.52% after exposure to toyocamycin, tebuconazole, and zhongshengmycin, respectively. However, the relative abundance of the second highest genera *Acinetobacter* did not show a significant difference among the four groups. Decreases in relative abundances under three types of antimicrobial treatment were observed in unclassified *Enterobacteriaceae*, *Pantoea*, *Aeromonas*, *Staphylococcus*, and *Ochrobactrum*. By contrast, the abundance of *Moraxellaceae* showed a significant increase in the antimicrobial treatment groups. Several genera changed differently in the three antimicrobial treatment groups. *Microbacterium* had a significant reduction during toyocamycin treatment, and *Pseudomonas* in the toyocamycin and tebuconazole groups also showed a decrease. *Chryseobacterium* had an increase in relative abundance in the tebuconazole and zhongshengmycin groups, and *Corynebacterium* had an increase in relative abundance in the toyocamycin and tebuconazole groups.

In the fungal community, the most dominant genera Hypocreales did not show significant difference in the four groups, although it constituted 83.85% of all the samples. After exposure to toyocamycin, tebuconazole, and zhongshengmycin, the relative abundance of unclassified k_fungi increased from 2.33 to 3.28, 8.11, 9.01%, respectively, whereas the relative abundance of Cutaneotrichosporon decreased from 5.97 to 3.22, 1.51, and 2.33%, respectively, after antimicrobial treatment. The relative abundance of *Talaromyces* had a different trend after antimicrobial treatment, showing a sharp increase in the toyocamycin and tebuconazole groups but a decreased in the zhongshengmycin group. *Vanrijia* had a low relative abundance, approximately 1% in all the samples. Therefore, antimicrobial treatment changed relative abundance in the samples, although the dominant genera did not show significant difference.

### Antimicrobial Treatment Altered the Community Structure of the Fat Body Microbiota in Brown Planthopper

**Figure 4:**
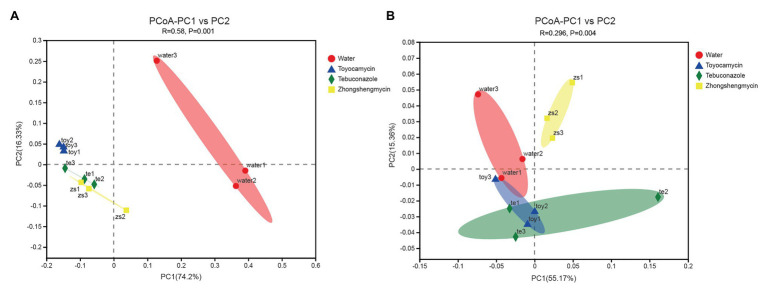
Comparative analysis of the microbial structures of the antibiotic treatment and control groups in *Nilaparvata lugens*. Principal coordinate analysis (PCoA) plots of bacterial **(A)** and fungal **(B)** unweighted UniFrac distances, which were obtained from Quantitative Insights into Microbial Ecology (QIIME).

The compositions of different microbial communities were compared on the basis of the beta diversity in the fat body of *N. lugens*. Beta diversity was visualized through PCoA. Community similarity was plotted based on Bray-Curitis and UniFrac metrics. For the bacterial communities, the two main coordinates PC1 and PC2 represented 74.2 and 16.33%, respectively. In Figure 4A, the PCoA results showed that the microbiota from different groups clustered separately from each other, and no overlap existed between the control and antimicrobial treatment groups (*p* = 0.001). The results suggested that the bacterial community structure was affected by antimicrobial treatment.

For the fungal community, the main two coordinates represented 55.17 and 15.36% of microbiota variation in the fat body (Figure 4B). The PCoA results showed that the control group clustered differently from the antimicrobial treatment group (with 999 permutations, ANOSIM, *p* = 0.001). After each antimicrobial treatment, the fungal communities from the fat body of control *N. lugens* clustered independently and distinctly from the antimicrobial treatment fat body samples (ANOSIM, *R* = 0.2963, *p* = 0.004).

### Antimicrobials Alter the Absolute Abundances of Bacterial and Fungal Communities

**Figure 5:**
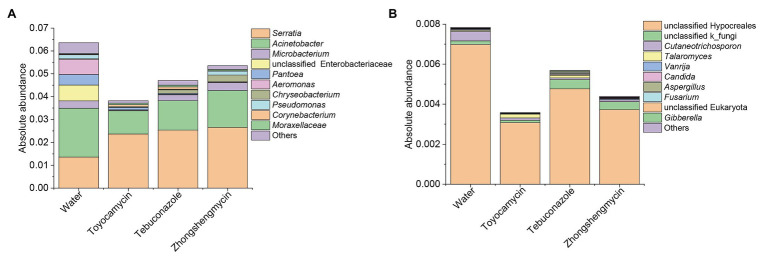
Absolute abundances of top 10 bacteria **(A)** and fungi **(B)** with respect to relative abundance at the genus level in BPH. Water, toyocamycin, tebuconazole, and zhongshengmycin represent treatment with water, toyocamycin, tebuconazole, and zhongshengmycin at recommended concentrations.

The absolute abundance of the bacteria and fungi were calculated through qPCR. The total bacterial and fungal abundances of the control group were higher than those of the antimicrobial treatment group (Figure 5). The toyocamycin group showed the lowest total abundance in the microbial community, followed by the tebuconazole group. The abundances of most strains of all genera decreased, particularly those of *Acinetobacter*, unclassified Enterobacteriaceae, *Pantoea*, and *Aeromonas*. Although total absolute abundance decreased under the antimicrobial exposure, the absolute abundance of *Serratia* increased upon antimicrobial treatment, indicating that *Serratia* is an important symbiont in the fat body and may have functions for resistance to these antimicrobials. In the fungal community, the absolute abundance of the dominant strain unclassified Hypocreales decreased after antimicrobial treatment. A similar trend was observed in the *Cutaneotrichosporon* group. However, the unclassified k_fungi increased in tebuconazole and zhongshengmycin group, indicating that the strain unclassified k_fungi may be resistant to the antimicrobials.

### Strains Disappear Upon Antimicrobial Treatment

Although dominant bacteria, such as *Acinetobacter*, *Serratia*, and unclassified Hypocreales, existed in the control and antimicrobial groups, several strains disappeared after antimicrobial treatment. Among the top 10 genera, the abundance of *Aeromonas* decreased from 10.35% in the control group to 0% in the three antimicrobial treatment groups. Other strains, such as *Wolbachia*, *Legionella*, and *Chromobacterium*, had high relative abundances in the control group but not detected after antimicrobial treatment. Some genera with extremely low abundances in the control group were killed in the antimicrobial treatment groups, including *Bacillus* subphylum Firmicutes, *Acidovorax*, *Exiguobacterium*, and *Lactobacillus*. *Bosea* disappeared in the toyocamycin and tebuconazole groups. As for the fungal community, no strain completely disappeared in the antimicrobial exposure group.

## Discussion

The insect fat body, mainly containing adipocytes, is the central tissue to store excess nutrition. The fat body participates in synthesis and decomposition of lipid, amino acids, protein, carbohydrate, and glycogen (Arrese and Soulages, 2010). The function of insect fat body has been proven by studying the transcriptomes of several pest species, such as the *Drosophila melanogaster* (Jiang et al., 2005), *Bombyx mori* (Cheng et al., 2006), and *Aedes aegypti* (Feitosa et al., 2006). However, for many insect species, the mycetocytes in the fat body, such as the whiteflies, aphids, and the BPH, are the key to utilize the low-nutrition sap from the plant (Douglas, 1989). The microbes provide essential compounds that the host could not produce alone and which may relate to the formation of biotypes, individual development and reproduction, resistance to pathogen infection, and detoxification metabolism (Xue et al., 2014; Bao and Zhang, 2019). Therefore, the microbes in the fat body play a critical role in its life cycle and interaction with plants.

The species of endosymbionts differed in different tissues, gender, fertilizing, and life stage. The microbes in the fat body of BPH mainly contained YLSs and bacterial symbionts. The changes of total endosymbionts in different developmental stage have been high-throughput sequenced, founding that Proteobacteria and Ascomycota were the most phylum of bacteria and fungi at all the developmental stages (Wang et al., 2020). The relative abundance of most dominant phylum Firmicutes and Actinobacteria differed between female and male BPHs (Wang et al., 2020). The comparison of bacterial symbionts in the ovary and fat body found the relative abundance of Firmicutes and Proteobacteria significantly differed in the different tissues (Zhang et al., 2019). The symbionts in the fertilized-eggs and non-fertilized eggs revealed that the abundance of Ascomycota decreased but the relative abundance of Basidiomycota increased after fertilizing (Shentu et al., 2020). In this study, we found that the Proteobacteria and Ascomycota are the most abundant in the fat body under all the groups after antimicrobial treatments, which is same as the result of all the developmental stages. For the top 10 abundance genera, seven genera among the top 10 abundance were belonged to Proteobacteria, including *Serratia*, *Acinetobacter*, unclassified Enterobacteriaceae, *Pantoea*, *Aeromonas*, *Pseudomonas*, and unclassified Moraxellaceae. Six genera among the top 10 abundance, such as unclassified Hypocreales, *Talaromyces*, *Candida*, *Aspergillus*, *Fusarium*, and *Gibberella* were part of Ascomycota. But the relative abundance of Proteobacteria increased in the toyocamycin treatment group but decreased in the tebuconazole and zhongshengmycin treatment groups compared with that in the control group. The relative abundance of Ascomycota had the same trend as Proteobacteria.

Because the fat body is the major tissue involved in nutrition metabolism and energy storage and release. Antimicrobials can disrupt nutrition supplication and affect survival rate. As indicated by genome analysis and metabolic pathway analysis, endosymbionts in the fat body induce complementary modifications in host genomes (Woolfit and Bromham, 2003; Xue et al., 2014). In this study, the relative abundance of several symbiotic strains decreased sharply after antimicrobial treatment, including *Wolbachia*, *Legionella*, *Chromobacterium*, unclassified Enterobacteriaceae, *Pantoea*, *Aeromonas*, *Staphylococcus*, *Ochrobactrum*, and *Cutaneotrichosporon*. These strains were identified as the obligate symbionts with the host and provided necessary metabolic pathways for the host. Thus, the inhibition of these strains may contribute to the dead of BPHs. For example, *Wolbachia* is the mutualistic bacterium in *Cimex lectularius* (Hosokawa et al., 2010), provides vitamin B, and increases resistance to pesticides. *Legionella* is mainly known as a significant obligate symbiont of insects located in the specialized organs of bacteriomes and supply some essential compounds to their hosts (Douglas, 1989; Říhová et al., 2017). Other strains with decreased abundance in the fat body were recognized as essential symbionts for insects and plants, including *Chromobacterium* (Martin et al., 2007a,b), *Pantoea* (Walterson and Stavrinides, 2015), *Aeromonas* (Lysyk et al., 2002), and *Staphylococcus*. The unclassified Enterobacteriaceae is a symbiont of aphids and imposes a variety of effects on its hosts, such as resistance to parasitoids and heat stress (Moran et al., 2005). *Ochrobactrum* is an alpha-proteobacterium and colonizes an entomopathogenic nematode *Acrobeloides maximus* guts and Ochrobactrum has mutualistic roles in the protection of entomopathogenic nematodes against pathogens (Baquiran et al., 2013). Thus, the decreases in these strains lead to the niche loss of symbionts in the fat body, which could be related to the mortality of BPH.

Change in bacterial abundance after antimicrobial treatment may due to adaptive responses and symbiont-mediated detoxification. When the rice plants sprayed with antimicrobials were fed to the BPH, the antimicrobials were absorbed by the gut and then entered the hemolymph, thus causing the stress of the microbial community in the fat body. Many studies focused on the effect of food on gut microbes and found that the diversity of microbiomes is related to diet composition (Yun et al., 2014; Chen et al., 2018; Xu et al., 2018, 2019). In this study, we found that antimicrobials in the diet can affect microbial community structure in the fat body. Our results further increase our knowledge of the influence of food on symbionts in insects. Treatment with fenitrothion enriched the fenitrothion-degrading strain *Burkholderia* in the gut, and the insect displayed resistance to fenitrothion in *Riptortus pedestris* (Kikuchi et al., 2012). In our study, the increased relative and absolute abundance of *Serratia* in the fat body of antimicrobial treatment groups suggested that *Serratia* is an important strain involved in resistance to antimicrobials. *Serratia* has been identified in many pests, including *Plutella xylostella* (Linnaeus) and *Dendroctonus genus* (Lin et al., 2014; Cano-Ramírez et al., 2016; Briones-Roblero et al., 2017; Hernández-García et al., 2018) and colonizes human intestinal organs as a nosocomial infection strain (Livrelli et al., 1996). It can express 16S rRNA methylase and thereby change the antimicrobial structure and lead to resistance to several antimicrobial agents, such as aminoglycosides and beta-lactam antimicrobials (Doi et al., 2004). In addition, *Serratia* can transfer antibiotic resistance ability to other strains through plasmid-mediated conjugation (Luisa et al., 2018). However, the function of *Serratia* remains unclear in BPH and needs further investigation. The abundances of *Moraxellaceae* and *Chryseobacterium* also increased after antimicrobial treatment. These strains are commonly found in insect gut and have functions in keratinous degradation (Gurav and Jadhav, 2013) and resistance to insecticides (Xia et al., 2013).

In this study, all antimicrobials were found harmful to BPH, as shown by decreased absolute microbe content and increased mortality. The antimicrobials can kill microbes, including useful microorganisms, leading to unbalance in the microbes’ ecological niche. However, the exact function of symbionts in the fat body of BPH remains unclear. Thus, we plan to conduct further studies on the functions of these bacteria and fungi to address the problems. Information on the effects of these fungicides and bactericides on the diversity of microbiome in the fat body of BPH will be useful in the integrated pest management of BPH.

## Data Availability Statement

The sequencing data have been uploaded in the NCBI, accession number PRJNA688202.

## Author Contributions

XS and XY: conceived and designed the experiments. JS: performed the experiments. XS and YS: analyzed the data and wrote the paper. All authors contributed to the article and approved the submitted version.

### Conflict of Interest

The authors declare that the research was conducted in the absence of any commercial or financial relationships that could be construed as a potential conflict of interest.

## Footnotes

^1^http://ccb.jhu.edu/software/FLASH
