# Supplementary material for: Antimicrobials Affect the Fat Body Microbiome and Increase the Brown Planthopper Mortality
Source: Front Physiol. 2021 Mar 15;12:644897. doi: 10.3389/fphys.2021.644897 (PMC8005595; doi:10.3389/fphys.2021.644897)

**Antimicrobials affect the fat body microbiome and increase brown planthopper mortality**

*Xuping Shentu^a^, Jiateng Shi^a^, Yang Song, Xiaoping Yu**

Zhejiang Provincial Key Laboratory of Biometrology and Inspection & Quarantine, College of Life Science, China Jiliang University, Hangzhou 310018, China

a: These authors contributed equally to this work

***Correspondence:** E-mail address: Prof. Xiaoping Yu; yxp@cjlu.edu.cn

**Figure S1** The plasmid standard curve of *Escherichia coli* 16s rDNA, *Saccharomyces cerevisiae* ITS and BPH β-actin sequence (A: BPH β-actin; B: *E. coli* 16s rDNA; C: *S. cerevisiae* ITS)


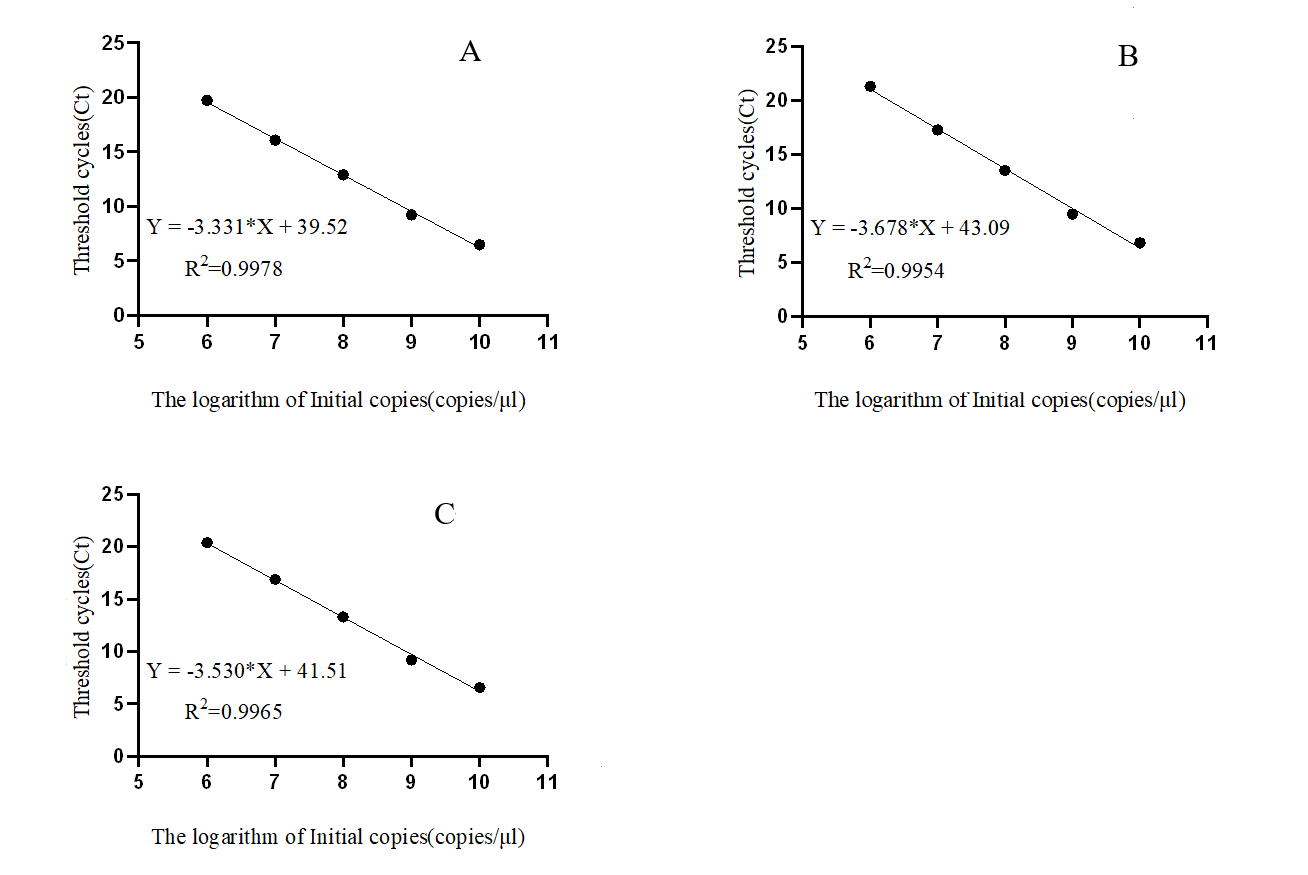


**Figure S2** The Venn figures of microbial community in the fat body of BPH


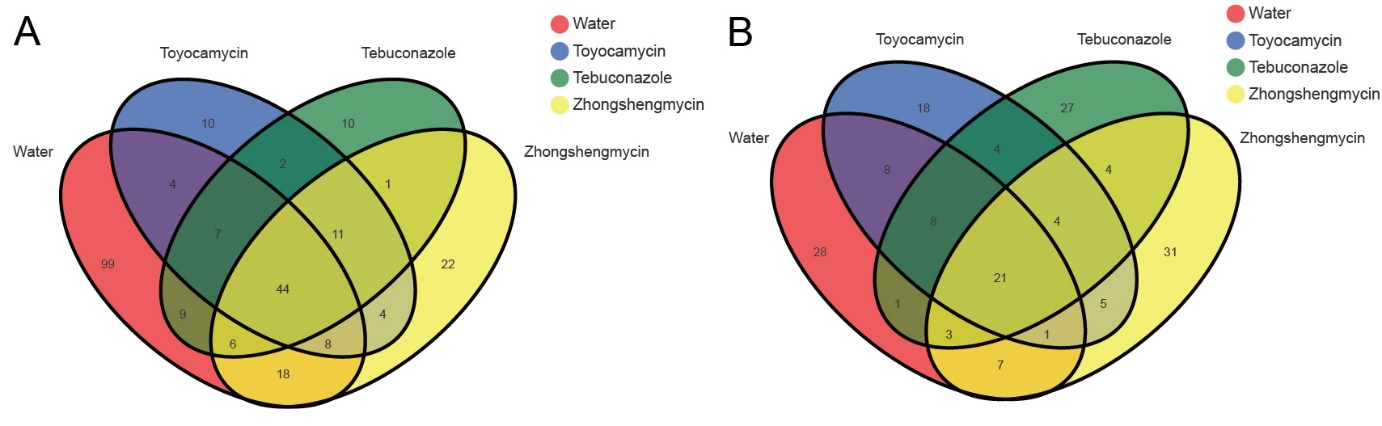

Supplement: Supplementary file 1 [file Data_Sheet_1.docx]
